# Effect of CO_2_ laser for the management of primary otosclerosis: A protocol for systematic review and meta-analysis

**DOI:** 10.1097/MD.0000000000020383

**Published:** 2020-05-29

**Authors:** Yi-ying Zhang, Jin-sheng Wang, Shu-hua Zhang, Gui-fang Liu, Peng-ju Zheng

**Affiliations:** a Department of Otolaryngology, Jiamusi University Affiliated Stomatological Hospital; b Department of Otorhinolaryngology, Second Hospital of Jiamusi Agricultural Reclamation; c Department of Otolaryngology, First Affiliated Hospital of Jiamusi University, Jiamusi, China.

**Keywords:** CO_2_ laser, effect, primary otosclerosis, safety

## Abstract

**Background:**

This study will explore the effect and safety of CO_2_ laser (COL) for the management of patients with primary otosclerosis (PO).

**Methods:**

The following electronic databases will be searched from inception to the present: PUBMED, EMBASE, The Cochrane Library, Web of Science, PsycINFO, Cumulative Index to Nursing and Allied Health Literature, Allied and Complementary Medicine Database, Chinese Biomedical Literature Database, VIP, WANGFANG, and China National Knowledge Infrastructure. No language limitation will be applied. All relevant randomized controlled trials using COL to treat patients with PO will be included. Two researchers will identify studies, collect data and evaluate the risk of bias of each included study independently. Any different views between 2 researchers will be resolved by a third researcher via discussion. Data analysis will be carried out using RevMan 5.3 software.

**Results:**

This study will evaluate the effect and safety of COL for the treatment of PO through hearing gain, tinnitus severity, incidence of intraoperative, health-related quality of life, other morbidities, and adverse events.

**Conclusion:**

This study will provide evidence for the effect and safety of COL in patients with PO.

**Study registration number:**

INPLASY202040110.

## 1 Introduction

Primary otosclerosis (PO) is a chronic hearing loss, which occurs due to abnormal bone growth in middle ear.^[1–5]^ It manifests with slowly progressive hearing loss, vertigo, and/or tinnitus.^[6–8]^ Several risk factors are associated with PO, including genetics, pregnancy, race, gender and age.^[6–10]^ Presently, no medications are available to treat PO. Although previous studies have reported that CO_2_ laser (COL) can benefit patients with PO, their results are inconsistent.^[11–21]^ Therefore, this systematic review aims to investigate the effect and safety of COL in patients with PO.

## 2 Methods and analysis

### 2.1 Study registration

This study protocol has been registered on INPLASY202040110. It has been reported in compliance with the guidelines of Preferred Reporting Items for Systematic review and Meta-Analysis (PRISMA) Protocols.^[22]^

### 2.2 Eligibility criteria

#### 2.2.1 Type of studies

Only randomized controlled trials (RCTs) on investigating the effect and safety of COL for the treatment of PO will be included in this study without limitations of language and publication status.

#### 2.2.2 Type of participants

We will include participants who were diagnosed as PO. There will be no limitations of race, gender, country, and disease duration.

#### 2.2.3 Type of interventions

In the experimental group, all participants underwent COT therapy in this study. COT combined with other modalities will be excluded.

In the control group, the comparators could be any management, except any forms of COT.

#### 2.2.4 Type of outcomes

Primary outcome measure is hearing gain, as assessed by pure-tone audiometry, or any other relevant tools.

Secondary outcome measurements are tinnitus severity, as measured by Tinnitus Functional Index or other indexes; incidence of intraoperative (bleeding and fractured footplate); health-related quality of life, as identified by Health-Related Quality-of-Life 14-Item Measure or other scales; and other morbidities (such as vomiting, vertigo, sensorineural hearing loss, and facial nerve paralysis).

### 2.3 Search strategy

We will search the following electronic databases from inception to the present: PUBMED, EMBASE, Cochrane Library, Web of Science, PsycINFO, Cumulative Index to Nursing and Allied Health Literature, Allied and Complementary Medicine Database, Chinese Biomedical Literature Database, VIP, WANGFANG, and China National Knowledge Infrastructure. We will not apply any limitations to language and publication status. The detailed search strategy of PUBMED is presented in Table 1. We will also build similar search strategies for other electronic databases.

**Table 1:**
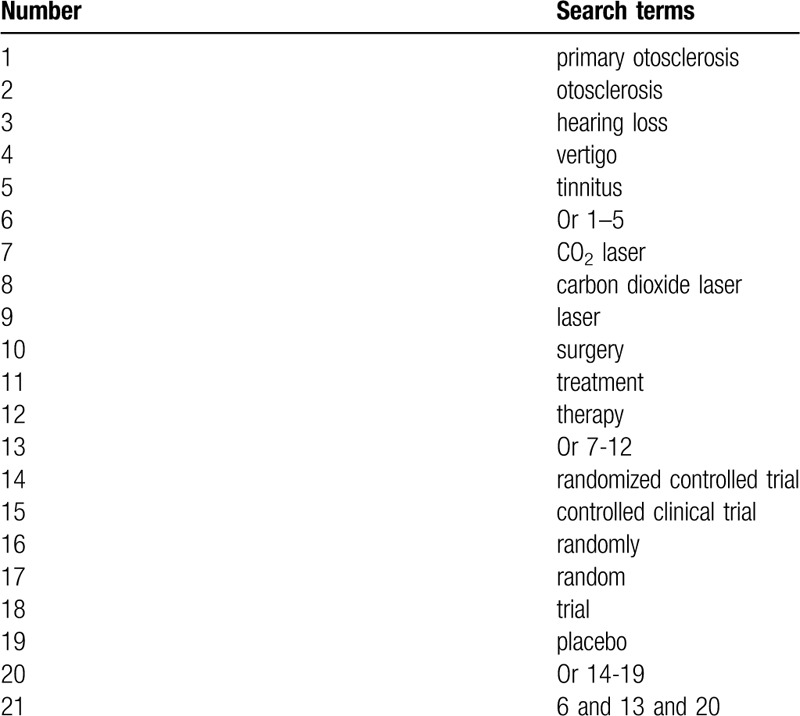
Search strategy sample of PUBMED.

We will also search grey literature to avoid missing any potential studies, including conference abstracts, ongoing trials from clinical trial registry, and reference lists of associated reviews.

### 2.4 Data collection and management

#### 2.4.1 Study selection

After literature search and duplicated studies removed, 2 independent researchers will work on the whole process of study selection according to the predefined inclusion criteria. Any discrepancies will be solved by discussion or consulting a third researcher. At the first stage, all titles and abstracts for potential studies will be scanned, and irrelevant studies will be removed. At the second stage, we will obtain full-texts of the remaining studies to further judge whether they meet all inclusion criteria. We will record reasons for all excluded studies. Details of the entire process of study selection will be presented in a PRISMA flowchart.

#### 2.4.2 Data collection

Data will be collected by 2 researchers independently with a pre-designed data collection form. It includes article information (such as first author, publication time, and location), patient information (such as sample size, race, sex, age, and types of PO), study design (such as detailed information of randomization, blind, and allocation), intervention and controls (such as types of therapies, duration, and frequency), outcomes (such as primary and secondary outcome measurements), safety, and sources of funding. If the data were unclear or missing in original study, we will contact corresponding authors to inquire it. If the entered RCTs include multiple groups, we will only extract data from groups, which is consistent with the objectives of this study. Any discrepancies between 2 researchers will be solved through team discussion with the help of a third researcher.

### 2.5 Assessment of risk of bias

Risk of bias will be checked by 2 independent researchers using Cochrane risk of bias tool for RCTs. It covers 7 items, and each item is classified as low, unclear, and high risk of bias. In case of divergences between 2 researchers, a third researcher will be involved to help resolve them by discussion.

### 2.6 Statistical analysis

RevMan 5.3 Software will be used for statistical analysis and for data analysis.

#### 2.6.1 Data synthesis

For continuous values, we will express them as mean difference with the same unit or standardized mean difference with different unit and 95% confidence intervals (CIs). For dichotomous values, risk ratio, and 95% CIs will be used to calculate them. *I*^2^ statistic test will be used for heterogeneity identification. It is interpreted as *I*^2^ ≤ 50% indicating minor heterogeneity, and we will use a fixed-effects model; while *I*^2^ > 50% showing obvious heterogeneity, and we will utilize a random-effects model. A meta-analysis will be carried out if heterogeneity is minor. Otherwise, we will conduct a subgroup analysis to explore the possible factors that may result in obvious heterogeneity. Additionally, we will carry out a descriptive summary and narrative synthesis.

#### 2.6.2 Subgroup analysis

We will conduct subgroup analysis according to the different characteristics of study or patient, different interventions or controls, and different outcome measurements.

#### 2.6.3 Sensitivity analysis

If necessary, we will perform sensitivity analysis to check the robustness of pooled outcome results by removing low quality studies.

#### 2.6.4 Reporting bias

If at least 10 eligible RCTs are included, we will undertake funnel plot and Egger regression test to check if there are possible reporting biases.^[22–23]^

### 2.7 Quality of evidence

All quality of evidence of each outcome will be identified using Grading of Recommendations Assessment Development and Evaluation (GRADE).^[24]^ It will be assessed through 5 fields, and each 1 is graded as high, moderate, low, or very low according to GRADE rating standards.

### 2.8 Ethics and dissemination

No ethical approval is sought, because all data utilized is in this study will be from published studies. This study is expected to be published on a peer-reviewed journal.

## 3 Discussion

Numerous clinical studies have reported the effect and safety of COL in patients with PO.^[9–20]^ However, we do not identify systematic review to assess the effect and safety of COL for the treatment of PO. This systematic review will search electronic databases and grey literatures to avoid missing eligible studies. The results of this study will provide most recent evidence to determine whether COL for the management of PO is effective or not.

## Author contributions

**Conceptualization:** Yi-ying Zhang, Shu-hua Zhang, Peng-ju Zheng.

**Data curation:** Yi-ying Zhang, Jin-sheng Wang, Gui-fang Liu, Peng-ju Zheng.

**Formal analysis:** Yi-ying Zhang, Jin-sheng Wang, Shu-hua Zhang, Gui-fang Liu.

**Funding acquisition:** Peng-ju Zheng.

**Investigation:** Peng-ju Zheng.

**Methodology:** Yi-ying Zhang, Jin-sheng Wang, Shu-hua Zhang, Gui-fang Liu.

**Project administration:** Peng-ju Zheng.

**Resources:** Yi-ying Zhang, Jin-sheng Wang, Shu-hua Zhang, Gui-fang Liu.

**Software:** Yi-ying Zhang, Jin-sheng Wang, Shu-hua Zhang, Gui-fang Liu.

**Supervision:** Peng-ju Zheng.

**Validation:** Yi-ying Zhang, Shu-hua Zhang, Peng-ju Zheng.

**Visualization:** Yi-ying Zhang, Jin-sheng Wang, Gui-fang Liu, Peng-ju Zheng.

**Writing – original draft:** Yi-ying Zhang, Jin-sheng Wang, Shu-hua Zhang, Gui-fang Liu, Peng-ju Zheng.

**Writing – review & editing:** Yi-ying Zhang, Shu-hua Zhang, Gui-fang Liu, Peng-ju Zheng.
